# Supplementary material for: GLI3 regulates muscle stem cell entry into GAlert and self-renewal
Source: Nat Commun. 2022 Jul 8;13:3961. doi: 10.1038/s41467-022-31695-5 (PMC9270324; doi:10.1038/s41467-022-31695-5)
Supplement: Supplementary file 1 — Supplementary Information [file 41467_2022_31695_MOESM1_ESM.pdf]

SUPPLEMENTARY INFORMATION

Primary Cilia Mediated-GLI3 Processing Regulates Muscle Stem Cell Entry into G_{Alert} and Self-Renewal

Caroline E. Brun[1,2], Marie-Claude Sincennes[1,2], Alexander Y.T. Lin[1,2], Derek Hall[1,2], William Jarassier[3], Peter Feige[1,2], Fabien Le Grand[3] and Michael A. Rudnicki[1,2,4,*]

[1] Sprott Centre for Stem Cell Research, Regenerative Medicine Program, Ottawa Hospital Research Institute, Ottawa, ON, K1H 8L6, Canada
[2] Department of Cellular and Molecular Medicine, Faculty of Medicine, University of Ottawa, Ottawa, ON, K1H 8M5, Canada
[3] Institut NeuroMyoGène, Pathophysiology and Genetics of Neuron and Muscle, Université Claude Bernard Lyon 1, CNRS UMR 5261, Inserm U1315, 69008 Lyon, France
[4] Department of Medicine, Faculty of Medicine, University of Ottawa, Ottawa, ON, K1H 8M5, Canada
* email: mrudnicki@ohri.ca

SUPPLEMENTARY FIGURES

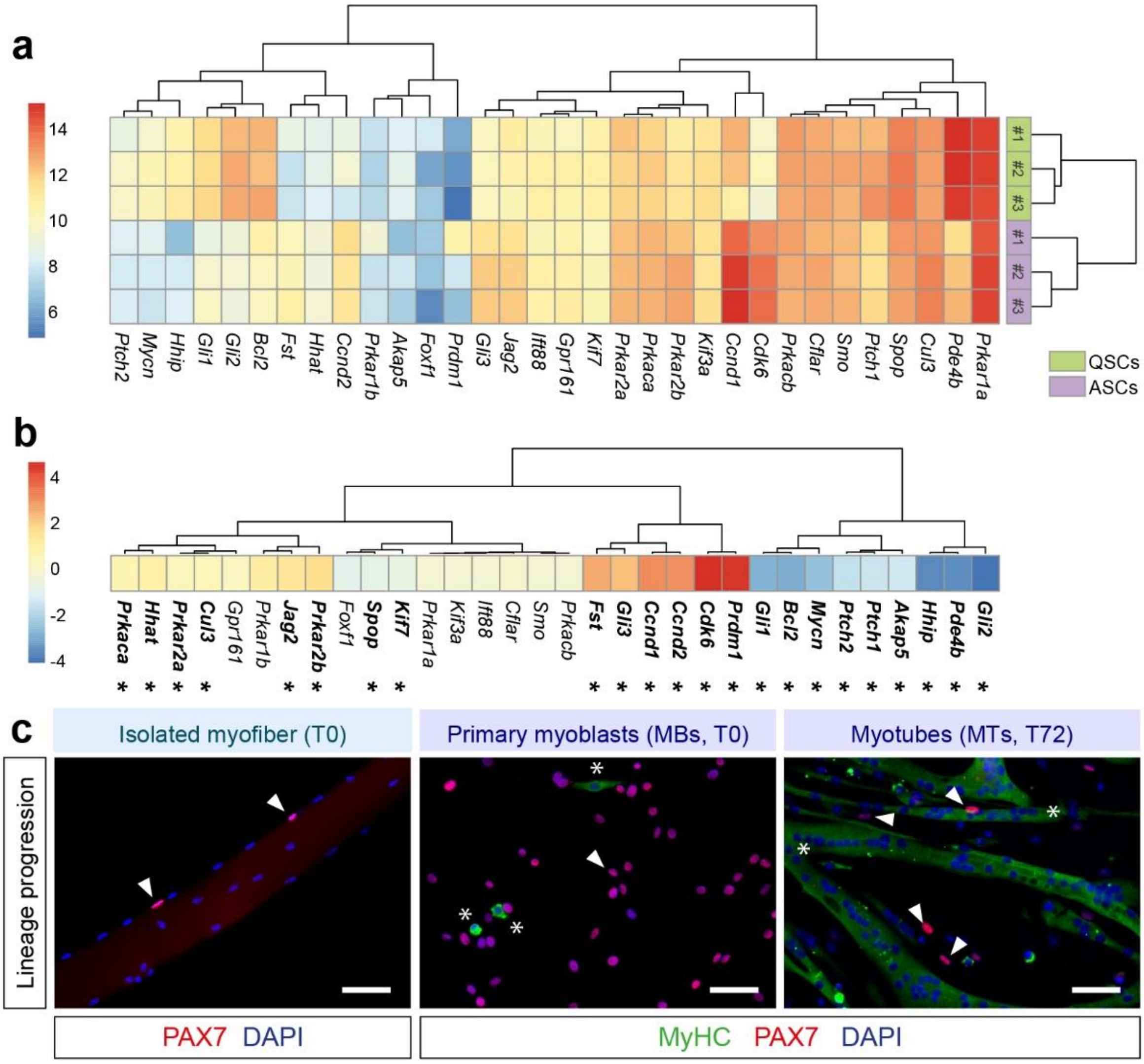

Supplementary Figure 1. Canonical Hedgehog downstream target genes are downregulated during satellite cell activation.

a) Heatmap from normalized and log2 transformed expression matrix of components and target genes of canonical Hedgehog signaling in quiescent (QSCs) and activated satellite cells (ASCs) (n = 3 males).

b) Heatmap showing fold-change values of canonical Hedgehog signaling genes in ASCs compared to QSCs. Significantly up- and down-regulated genes are written in bold with a star (*) that indicates *p* adjusted values (padj) < 0.05 (Wald test followed by multiple testing correction using the Benjamini–Hochberg method to control the false discovery rate).

c) Representative immunofluorescence staining of PAX7 (red) labelling quiescent satellite cells on an isolated myofiber (T0), and primary myoblasts (MBs, T0) and reserve cells appearing along the myotubes (MTs, T72). Myosin heavy chain (MyHC, green, asterisks) labels differentiated muscle cells or myotubes. Nuclei are stained with DAPI (blue). Scale bars, 10µm.

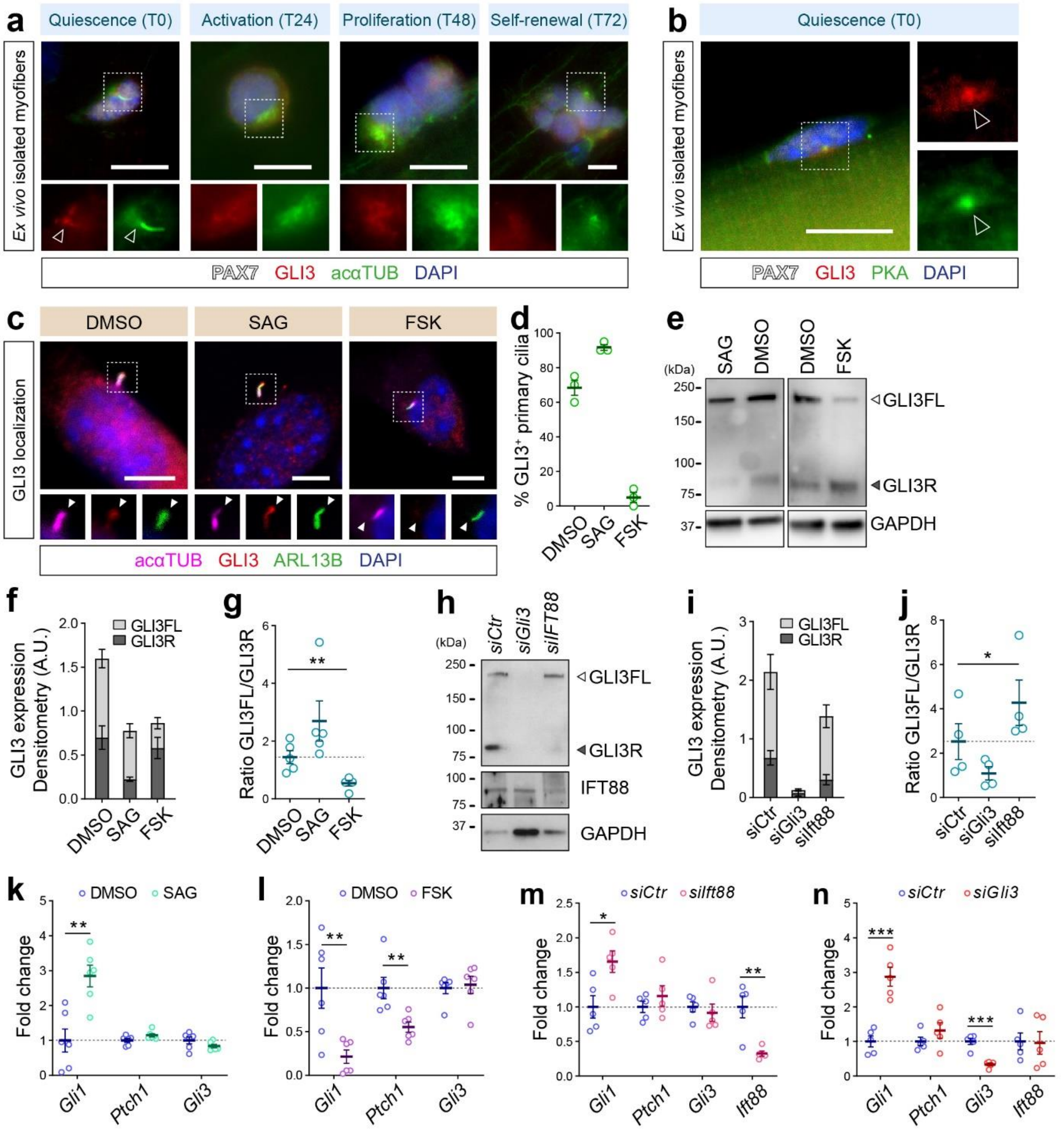

Supplementary Figure 2. GLI3 processing and subcellular localization relies on the primary cilium and regulates Hedgehog signaling target genes.

a) Representative immunostaining showing GLI3 (red) localization in PAX7+ (white) quiescent (T0), activated (T24) and proliferating (T48-72) satellite cells on isolated and cultured myofibers. Acetylated α-TUBULIN (acαTUB, green) stains the primary cilium (T0) and the microtubule-organizing centers (T24-72). Nuclei are stained with DAPI (blue). Scale bars, 10µm.

b) Immunostaining showing GLI3 (red) and PKA (green) localization in a PAX7+ (white) quiescent satellite cell on freshly isolated myofibers. PKA (green) localizes at and around the centrosome areas. Scale bars, 5µm.

c) Representative immunofluorescence pictures showing GLI3 (red) localization in primary cilia stained with both acαTUB (purple) and ARL13B (green) upon SAG (Smoothened agonist) and FSK (forskolin) treatment. DMSO (vehicle) is used as a control. Scale bars, 5µm.

d) Proportion of DMSO, SAG or FSK treated-myoblasts that exhibit GLI3 staining in the primary cilium (n = 3 biologically independent samples).
e) Immunoblot analysis of GLI3 full-length (GLI3FL) and repressor (GLI3R) in DMSO, SAG or FSK treated-myoblasts. GAPDH is used as a loading control.
f) Densitometric analysis of the level of GLI3FL (light gray) and GLI3R (dark gray) relative to GAPDH signals of 5 biological replicates.
g) Ratio of GLI3FL/GLI3R relative to GAPDH (n = 5 biological replicates).
h) Immunblot analysis of GLI3 and IFT88 showing GLI3FL and GLI3R levels upon efficient *Gli3* and *Ift88* knockdown in primary myoblasts.
i) Densitometric analysis of the level of GLI3FL (light gray) and GLI3R (dark gray) relative to GAPDH signals of 4 biological replicates.
j) Ratio of GLI3FL/GLI3R relative to GAPDH (n = 4 biological replicates).
k) Expression of *Gli1* and *Ptch1*, two Hedgehog target genes, normalized to *Ppia* and *Gapdh*, upon SAG treatment (n = 6 biologically independent samples).
l) Expression of *Gli1* and *Ptch1* normalized to *Ppia* and *Gapdh*, upon FSK treatment (n = 6 biologically independent samples).
m) Expression of *Gli1*, *Ptch1*, *Gli3* and *Ift88* normalized to *Ppia* and *Gapdh* in primary myoblasts, 48h after treatment with siRNA for *Ift88* (*siIft88*) or with a non-target control (*siCtr*) (n = 5 biologically independent samples).
n) Expression of *Gli1*, *Ptch1*, *Gli3* and *Ift88* normalized to *Ppia* and *Gapdh* in primary myoblasts, 48h after treatment with siRNA for *Gli3* (*siGli3*) or with a non-target control (*siCtr*) (n = 5 biologically independent samples).
Unless otherwise indicated, scale bars, 10µm; Means ± SEM; Two-tailed paired t test (**g**), One-way ANOVA test with Fisher's LSD for multiple comparisons (**j**), Multiple unpaired t tests with Welch's correction (**k-m**); *$p < 0.5$, **$p < 0.01$, ***$p < 0.001$.

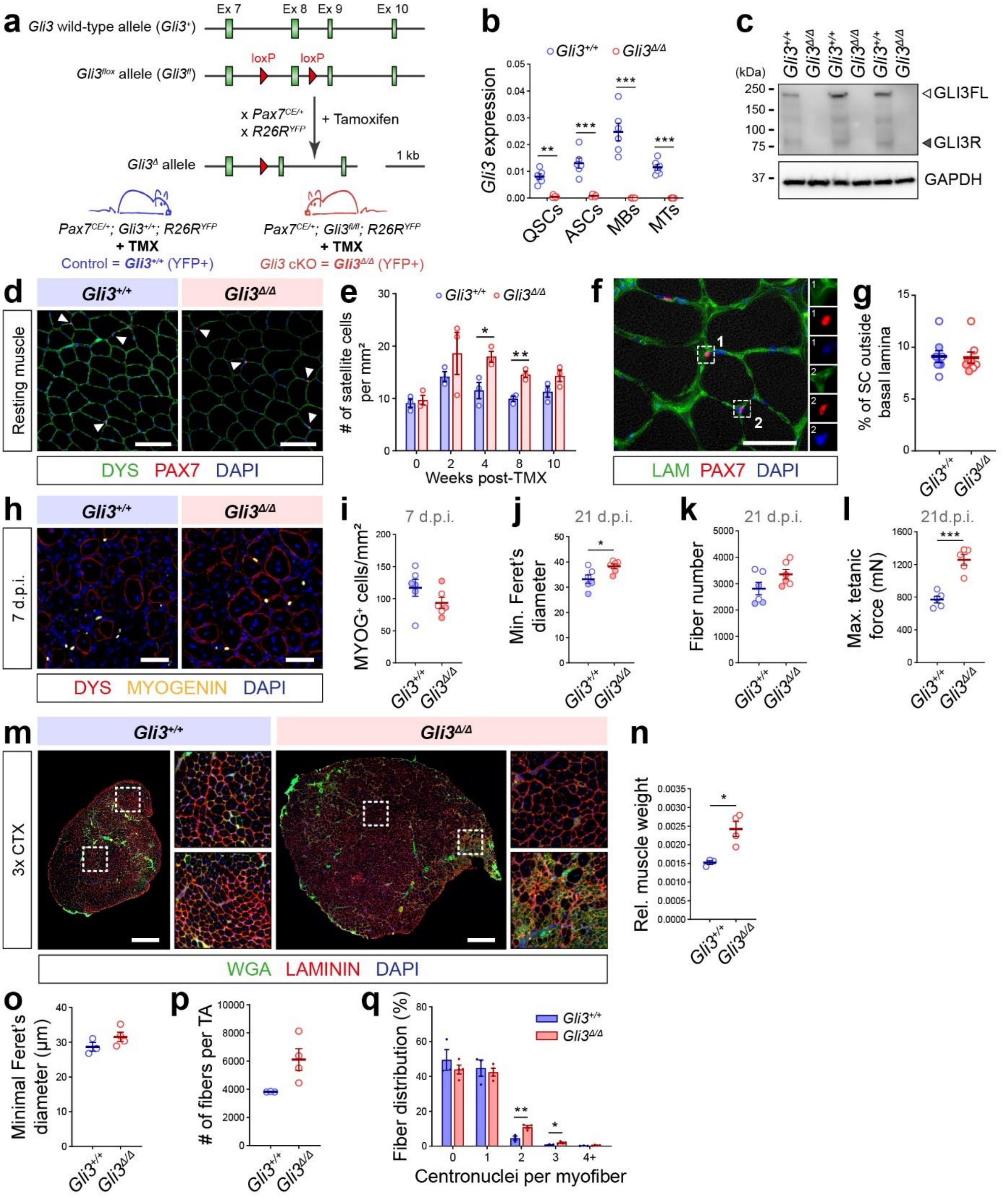

Supplementary Figure 3. Phenotype of *Gli3*$^{+/+}$ and *Gli3*$^{\Delta/\Delta}$ muscle following a single and triple cardiotoxin-injuries.

a) The exon 8 of the *Gli3* floxed allele (*Gli3*^{fl}) is flanked by two loxP sites, allowing for its tamoxifen (TMX)-inducible recombination by the CreER recombinase. The CreER inserted downstream the *Pax7* stop codon (*Pax7*$^{CE/+}$) allows endogenous PAX7 expression while permitting specific ablation of *Gli3* in satellite cells upon TMX treatment. In addition, the *R26R*^{YFP} allele was added allowing for tracing the satellite cell (SC) population. TMX-treated *Pax7*$^{CE/+}$; *Gli3*$^{+/+}$; *R26R*^{YFP} mice are used as control (*Gli3*$^{+/+}$) while TMX-treated *Pax7*$^{CE/+}$; *Gli3*$^{fl/fl}$; *R26R*^{YFP} mice where SCs will be conditionally knocked-out for *Gli3* are referred as *Gli3*$^{\Delta/\Delta}$.

b) Expression analysis by RT-qPCR of *Gli3* normalized to *Ppia* and *Rps18* in quiescent (QSCs) and activated (ASCs) satellite cells, primary myoblasts (MBs), and 3 days differentiated myotubes (MTs) (n = 6 biologically independent samples).
c) Western blotting for GLI3 confirming the knockout efficiency in primary myoblasts. GAPDH is used as a loading control (n = 3 biological samples).
d) Representative immunofluorescence picture showing transversal cross-sections of resting TA muscle in *Gli3*$^{+/+}$ and *Gli3*$^{\Delta/\Delta}$ mice. PAX7 (red) labels the satellite cells and DYS (green) delineates the myofibers. Nuclei are stained with DAPI (blue). Scale bars represent 50µm.
e) Quantification of the number of PAX7^{+} satellite cells per mm² in resting TA muscle from *Gli3*$^{+/+}$ and *Gli3*$^{\Delta/\Delta}$ mice following tamoxifen treatment (n = 3 males).
f) Immunofluorescence for PAX7 (red) and LAMININ (LAM, green) showing 1) a satellite cell within its niche and 2) a satellite cell surrounded by the basal lamina outside its niche.
g) Proportion of satellite cells found outside the basal lamina in *Gli3*$^{+/+}$ and *Gli3*$^{\Delta/\Delta}$ resting TA muscles.
h) Immunostaining of MYOGENIN (yellow) at 7 d.p.i. showing the differentiated muscle cells and DYSTROPHIN (DYS, red) delineating the regenerating myofibers. DAPI stains the nuclei (blue).
i) Quantification of MYOG^{+} cells per mm² TA section of all *Gli3*$^{+/+}$ and *Gli3*$^{\Delta/\Delta}$ mice at 7 d.p.i.
j) Minimal Feret's diameter of all *Gli3*$^{+/+}$ and *Gli3*$^{\Delta/\Delta}$ mice at 21 d.p.i.
k) Number of myofibers per TA section of all *Gli3*$^{+/+}$ and *Gli3*$^{\Delta/\Delta}$ mice at 21 d.p.i.
l) Maximum tetanic force of TA muscles of *Gli3*$^{+/+}$ and *Gli3*$^{\Delta/\Delta}$ mice at 21 d.p.i. (n = 5 males).
m) Representative immunofluorescence picture of *Gli3*$^{+/+}$ or *Gli3*$^{\Delta/\Delta}$ regenerated muscle following a triple injury (3x CTX). LAMININ (red) and WGA (green) delineate the myofibers. DAPI stains the nuclei.
n) TA muscle weight normalized to total body weight of *Gli3*$^{+/+}$ and *Gli3*$^{\Delta/\Delta}$ mice following 3x CTX.
o) Minimal Feret's diameter of regenerated myofibers of 3x CTX *Gli3*$^{+/+}$ and *Gli3*$^{\Delta/\Delta}$ mice.
p) Number of myofibers per TA section of *Gli3*$^{+/+}$ and *Gli3*$^{\Delta/\Delta}$ mice after 3x CTX.
q) Distribution of regenerated myofibers according to their number of centrally located nuclei (centronuclei) of 3x CTX *Gli3*$^{+/+}$ and *Gli3*$^{\Delta/\Delta}$ mice.
Means ± SEM; ANOVA test with Fisher's LSD for multiple comparisons (**b**), Multiple unpaired t tests with Welch's correction (**e**, **j**, **l**, **n**, **q**); $*p < 0.5$, $**p < 0.01$, $***p < 0.001$.
For the single injury, unless otherwise indicated, n = 6 (3 males, empty dots, and 3 females, colored dots, for each genotype); Scale bars, 50µm.
For the triple injuries (3x CTX), n = 3 *Gli3*$^{+/+}$ and 4 *Gli3*$^{\Delta/\Delta}$ males; Scale bars, 500µm.

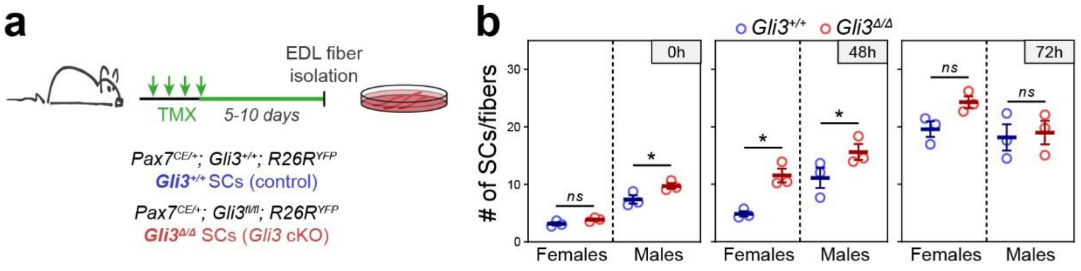

Supplementary Figure 4. *Gli3* deletion enhances satellite cell proliferation on isolated myofibers.

a) Experimental design. Single EDL myofibers are isolated from tamoxifen-treated *Pax7*$^{CE/+}$; *Gli3*$^{+/+}$; *R26R*^{YFP} (control, *Gli3*$^{+/+}$) and *Pax7*$^{CE/+}$; *Gli3*$^{fl/fl}$; *R26R*^{YFP} (*Gli3* conditional knockout, *Gli3*$^{\Delta/\Delta}$) mice and cultured for 48h and 72h to follow satellite cell proliferation and differentiation.

b) Quantification of the number of satellite cells per myofiber immediately after isolation (0h), or after 48h and 72h of culture (n = 3 males and 3 females for each genotype). Means ± SEM; One-way ANOVA test with Fisher's LSD for multiple comparisons; *$p < 0.5$.

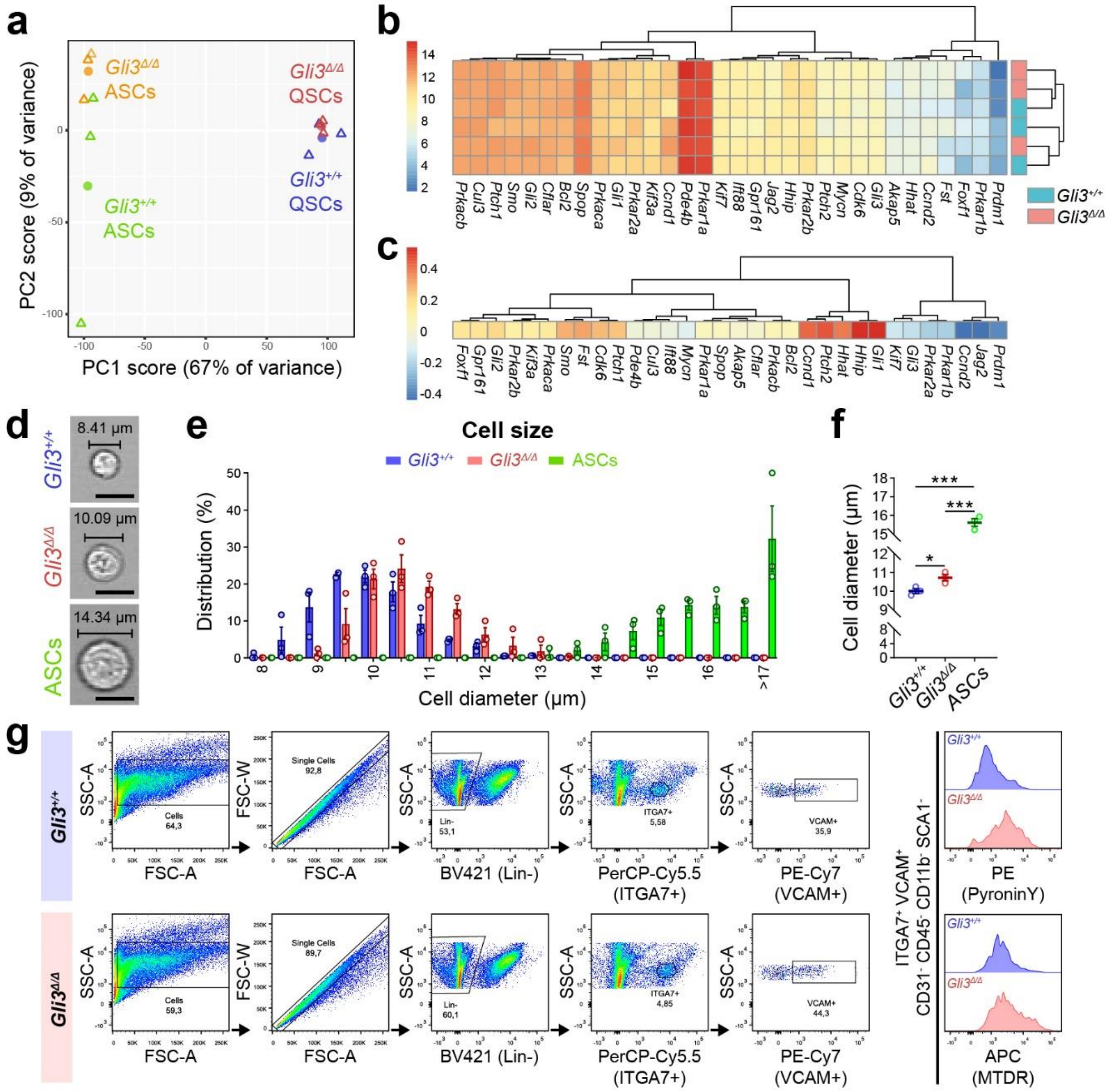

Supplementary Figure 5. ***Gli3*** **deletion induces satellite cell transition to** $\mathbf{G_{Alert}}$**.**

a) Principal component analysis (PCA) of global transcriptomes of *Gli3*$^{+/+}$ and *Gli3*$^{\Delta/\Delta}$ quiescent satellite cells (QSCs) and activated satellite cells (ASCs). Each triangle represents a biological replicate. Each dot represents the mean of 3 biological samples (n =3 males for each condition and genotype).

b) Heatmap from normalized and log2 transformed expression matrix of components and target genes of canonical Hedgehog signaling in *Gli3*$^{\Delta/\Delta}$ and *Gli3*$^{+/+}$ QSCs (n = 3 males).

c) Heatmap showing fold change values of canonical Hedgehog signaling genes in *Gli3*$^{\Delta/\Delta}$ compared to *Gli3*$^{+/+}$ QSCs.

d) Representative images of freshly sorted *Gli3*$^{+/+}$ and *Gli3*$^{\Delta/\Delta}$ quiescent satellite cells (QSCs) and *Gli3*$^{+/+}$ activated satellite cells (ASCs).

e) Distribution of *Gli3*$^{+/+}$ QSCs, *Gli3*$^{\Delta/\Delta}$ QSCs and *Gli3*$^{+/+}$ ASCs according to their diameter (n = 3 males, >100 cells analyzed per mouse).

f) Mean cell diameter of *Gli3*$^{+/+}$ QSCs, *Gli3*$^{\Delta/\Delta}$ QSCs and *Gli3*$^{+/+}$ ASCs (n = 3 males, >100 cells analyzed per mouse, source data are provided in the Source Data file).

g) Flow cytometry gating strategy used to analyze PyroninY and MitoTracker Deep Red (MTDR) staining in QSCs from *Gli3*$^{+/+}$ and *Gli3*$^{\Delta/\Delta}$ mice.

Means ± SEM; One-way ANOVA test with Fisher's LSD for multiple comparisons; **p* < 0.5, ***p* < 0.01, ****p* < 0.001.

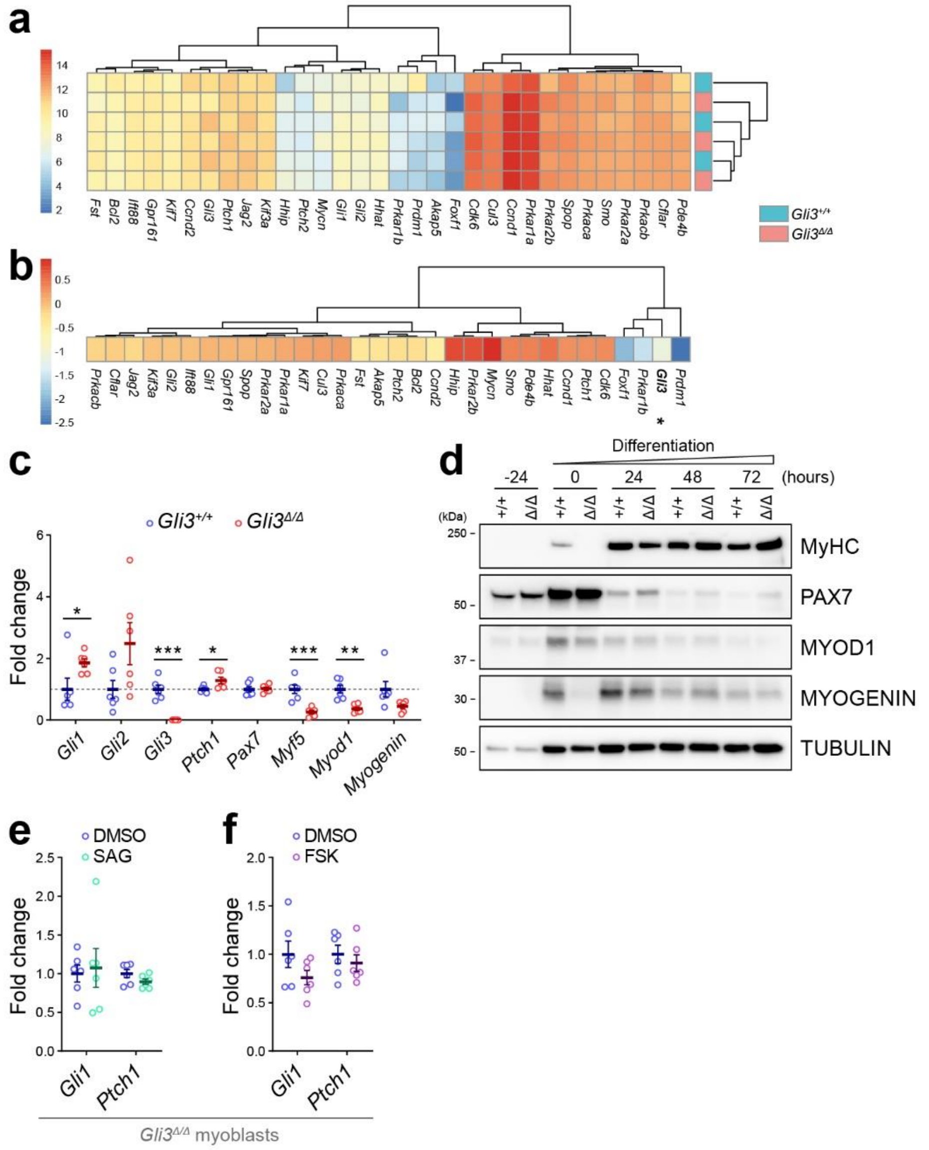

Supplementary Figure 6. *Gli3* deletion increases satellite cell proliferation *in vivo*.

a) Heatmap from normalized and log2 transformed expression matrix of components and target genes of canonical Hedgehog signaling in *Gli3*$^{\Delta/\Delta}$ and *Gli3*$^{+/+}$ ASCs (n = 3 males).

b) Heatmap showing fold change values of canonical Hedgehog signaling genes in *Gli3*$^{\Delta/\Delta}$ compared to *Gli3*$^{+/+}$ ASCs. Only *Gli3* (in bold, with a star) is significantly down-regulated with a padj < 0.05 (Wald test followed by multiple testing correction using the Benjamini–Hochberg method to control the false discovery rate).

c) RT-qPCR analysis of *Gli1-3*, *Ptch1*, *Pax7*, *Myf5*, *Myod1* and M*yogenin* normalized to *Ppia* and *Gapdh* in *Gli3*$^{\Delta/\Delta}$ and *Gli3*$^{+/+}$ proliferating primary myoblasts (n = 3 males and 3 females for each genotype)).

d) Immunoblot analysis of MyHC, PAX7, MYOD1 and MYOGENIN from *Gli3*$^{+/+}$ and *Gli3*$^{\Delta/\Delta}$ myoblasts differentiated for 72h. TUBULIN is used as a loading control.

e) Expression of *Gli1* and *Ptch1* normalized to *Ppia* and *Gapdh*, in *Gli3*$^{\Delta/\Delta}$ myoblasts upon SAG treatment (n = 6 biologically independent samples).

f) Expression of *Gli1* and *Ptch1* normalized to *Ppia* and *Gapdh*, in *Gli3*$^{\Delta/\Delta}$ myoblasts upon FSK treatment (n = 6 biologically independent samples).

Means ± SEM; Multiple unpaired t tests with Welch's correction; *$p < 0.05$; **$p < 0.01$; ***$p < 0.001$

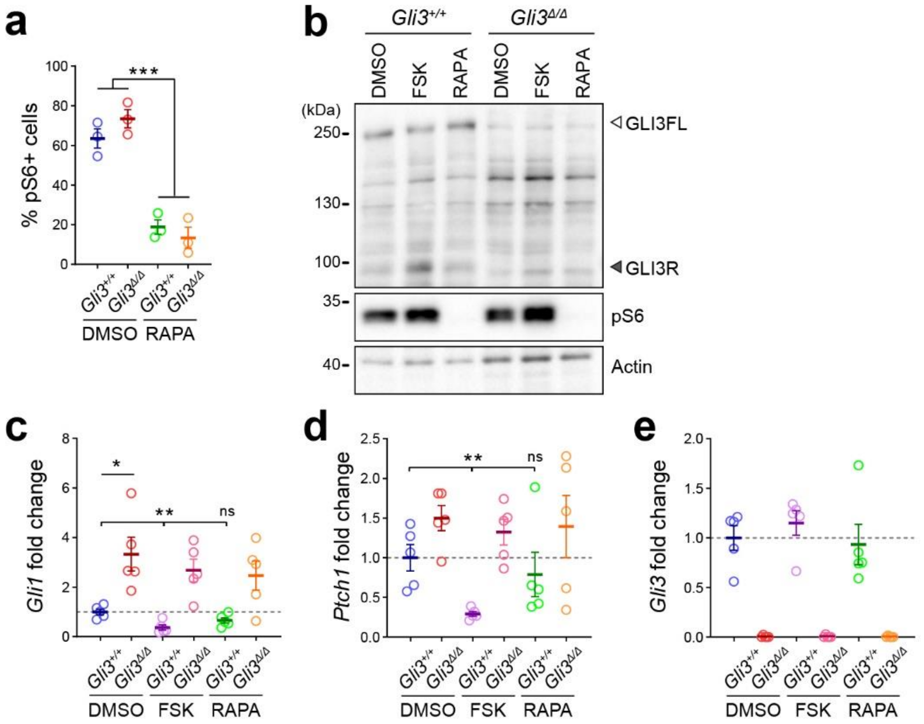

Supplementary Figure 7. GLI3 modulates mTORC1 signaling in muscle cells.

a) Proportions of phospho-S6$^+$ (pS6$^+$) cells from *Gli3*$^{+/+}$ and *Gli3*$^{\Delta/\Delta}$ myoblasts upon FSK and RAPA treatments (n = 3 biological replicates).

b) Immunoblot analysis of GLI3 full-length (GLI3FL) and repressor (GLI3R) and phospho-S6 (pS6) in DMSO, FSK or RAPA treated-myoblasts. ACTIN is used as a loading control.

c) Expression level of *Gli1* and **d**) and *Ptch1*, two canonical Hedgehog target genes, normalized to *Ppia* and *Hprt*, upon FSK and RAPA treatments (n = 5 biological replicates).

e) Expression level of *Gli3* normalized to *Ppia* and *Hprt*, upon FSK and RAPA treatments (n = 5 biological replicates).

Means ± SEM; ; One-way ANOVA test with Fisher's LSD for multiple comparisons; *$p < 0.05$; **$p < 0.01$; ***$p < 0.001$

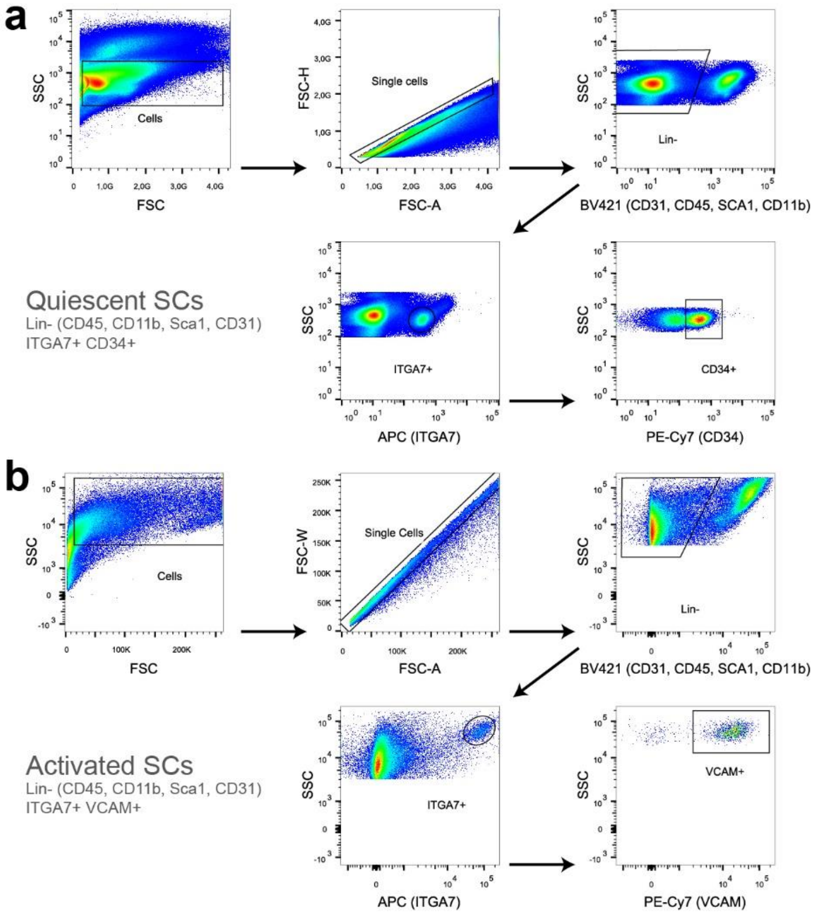

Supplementary Figure 8. Gating strategy for quiescent and activated satellite cell sort.

a) Single cell suspensions were obtained from all hindlimb muscles. Cell doublets were excluded from the analysis (FSC-A *vs* FSC-H). Satellite cells (CD31-, CD45-, SCA1-, CD11b-, ITGA7+, CD34+) were sorted and used for analysis.

b) Satellite cell activation is induced by intramuscular injections of cardiotoxin in *tibialis anterior* (TA) and *gastrocnemius* (GA) muscles of both legs. 3 days post-injury, single cell suspensions are obtained from TA and GA injured muscles. Cell doublets are excluded from the analysis (FSC-A *vs* FSC-W). Satellite cells (CD31-, CD45-, SCA1-, CD11b-, ITGA7+, VCAM+) are sorted and used for analysis.

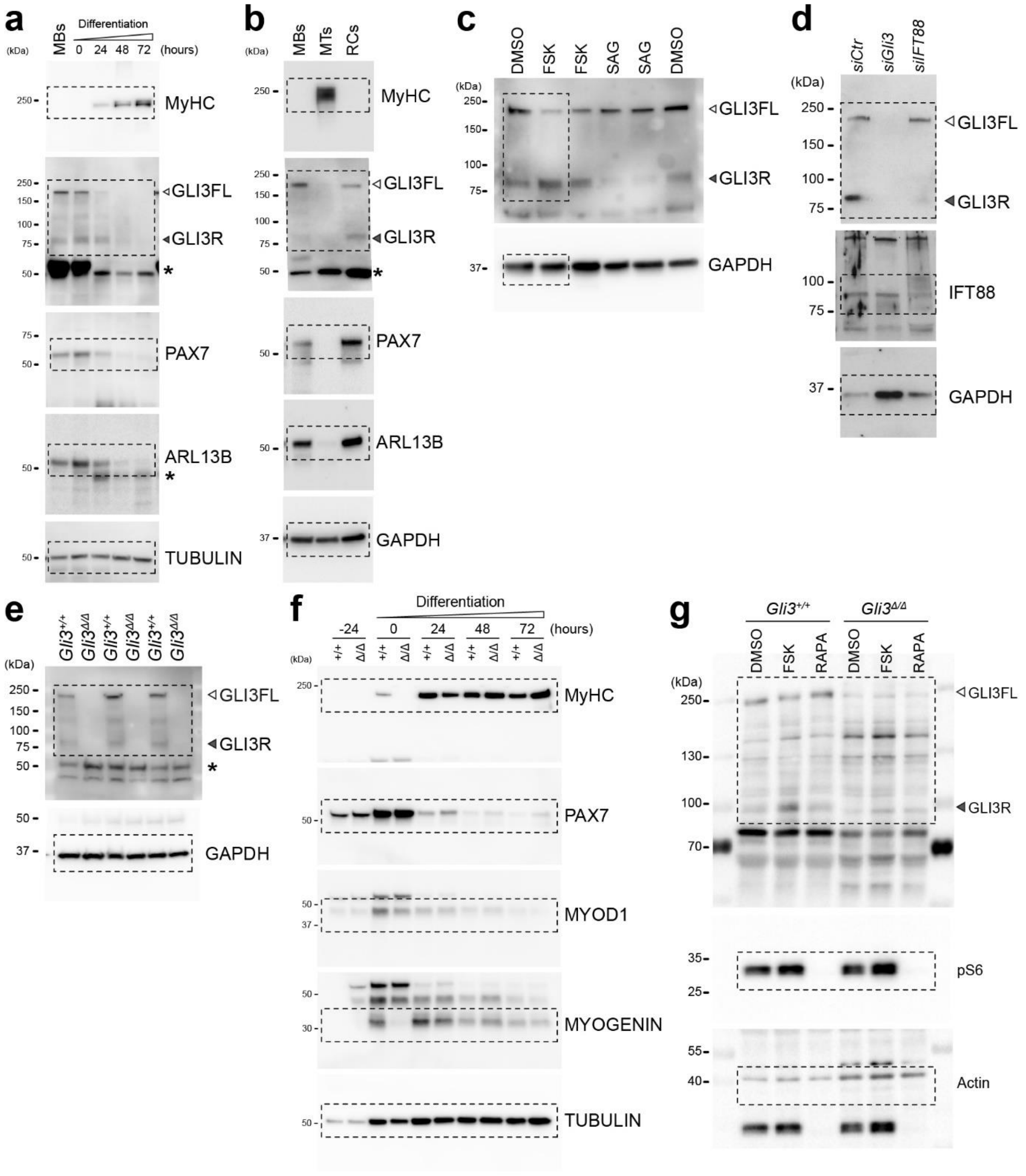

Supplementary Figure 9. Full scans of immunoblots.

a) Figure 2c

b) Figure 2d

c) Supplementary Figure 2e

d) Supplementary Figure 2h

e) Supplementary Figure 3c

f) Supplementary Figure 6d

g) Supplementary Figure 7b

SUPPLEMENTARY TABLES

Supplementary Table 1. List of antibodies.

**Primary Antibody**	**Company**	**Reference**	**Dilution** (IF)	**Dilution** (WB)
Mouse anti-ARL13B [N295B/66]	Abcam	ab136648	1/500	1/500
Mouse anti-α-TUBULIN (acetyl K40) [6-11B-1]	Abcam	ab11323	1/1000	N/A
Mouse anti-PAX7	DSHB	PAX7	1/2	1/10
Mouse anti-GLI3 [6F5]	Gift from Dr. S. Scales (Genentech)	N/A	N/A	1/250
Goat anti-GLI3	R&D Systems	AF3690	1/300	1/500
Rabbit anti-PKA R2/PKR2 (phospho S99) [E151]	Abcam	ab238951	1/250	N/A
Rabbit anti-IFT88	Proteintech	13967-1-AP	N/A	1/500
Mouse anti-TUBULIN (clone DM1A)	Sigma-Aldrich	T9026	N/A	1/10000
Mouse anti-GAPDH	UBC AbLab	21-0017	N/A	1/10000
Mouse anti-alpha-ACTIN	Santa Cruz	sc-32251	N/A	1/5000
Mouse anti-Myosin Heavy Chain (MyHC)	DSHB	MF20	1/2000	1/1000
Mouse anti-MYOD1 [5.8A]	Agilent Dako	M3512	N/A	1/2500
Mouse anti-MYOGENIN (clone F5D)	Santa Cruz	sc-12732	1/500	1/2000
Rabbit anti-DYSTROPHIN	Abcam	ab15277	1/1000	N/A
Rat anti-LAMININ [4H8-2]	Sigma-Aldrich	L0663	1/1000	N/A
Rabbit anti-LAMININ	Sigma-Aldrich	L9393	1/1000	N/A
Chicken anti-SYNDECAN-4	Gift from Dr. B. Olwin	N/A	1/1000	N/A
Chicken anti-GFP (YFP)	Abcam	ab13970	1/1000	N/A
Rabbit anti-GFP (YFP)	ThermoFisher Scientific	A-11122	1/1000	1/2000
Rabbit anti-phospho-S6 Ribosomal Protein (Ser235/236) [D57.2.2E]	Cell Signaling Technology	4858	1/100	1/2500
Alexa647 mouse anti-Integrin alpha7 (clone R2F2)	UBC AbLab	67-0010-10	1/100	N/A
Mouse anti-Integrin alpha7-Biotin (clone 3C12)	Miltenyi Biotec	130-102-125	1/100	N/A
Mouse anti-CD34-Biotin (clone REA383)	Miltenyi Biotec	130-105-830	1/100	N/A
BV421 mouse anti-SCA1 (clone D7)	BD Biosciences	553108	1/500	N/A
BV421 mouse anti-CD45 (clone 30-F11)	BD Biosciences	12-0451-83	1/500	N/A
BV421 mouse anti-CD31 (clone 390)	BD Biosciences	12-0311-81	1/500	N/A
BV421 mouse anti-CD11b (clone M1/70)	BD Biosciences	12-0112-81	1/500	N/A
PE-Cy7 mouse anti-CD106 (VCAM1)	BioLegend	105719	1/100	N/A
PerCP/Cyanine5.5 Streptavidin	BioLegend	405214	1/500	N/A

Supplementary Table 2. List of primers.

Gene	Forward	Reverse
Gli1	CCCTTTCTTGAGGTTGGGAT	CCCAGACGGCGAGACAC
Gli2	CCCATGACTCTCACCTCCAT	CTTGACCTTGCTCCGCTTAT
Gli3	TAGCTTCGACCTTCAGACCA	AGGGGTAGGTGAAGCTCAAT
Ift88	GGAAAATGTTCATCTGGCACC	CTGACTGCTGTGCTTGGTAT
Ptch1	TTCTGCTGCCTGTCCTCTTA	GCAAACCGGACGACACTT
Myf5	TGACGGCATGCCTGAATGTA	ATCTGCAGCACATGCATTTGATA
Myod1	TAGTAGGCGGTGTCGTAGCC	TACAGTGGCGACTCAGATGC
Myogenin	CAACCCAGGAGATCATTTG	CATATCCTCCACCGTGAT
Pax7	GACGACGAGGAAGGAGACAA	ACATCTGAGCCCTCATCCAG
Rps18	AACGGTCTAGACAACAAGCTG	AGTGGTCTTGGTGTGCTGAC
Gapdh	TGTGTCCGTCGTGGATCTGA	CCTGCTTCACCACCTTCTTGA
Hprt	GGCCAGACTTTGTTGGATTTG	CACAGGACTAGAACACCTGC
Ppia	CAGTGCCAAGACTGAATG	GTCGGAAATGGTGATCTT

INVENTORY OF SUPPORTING INFORMATION

Supplementary Data file 1. Fold change analysis of transcriptomes from *Gli3*$^{+/+}$ ASCs vs *Gli3*$^{+/+}$ QSCs.

Supplementary Data file 2. Fold change analysis of transcriptomes from ASCs *Gli3*$^{\Delta/\Delta}$ vs *Gli3*$^{+/+}$ QSCs (QSC FC Gli3cKO vs Gli3Ctr) and *Gli3*$^{\Delta/\Delta}$ vs *Gli3*$^{+/+}$ ASCs (ASC FC Gli3cKO vs Gli3Ctr).
