# Supplementary material for: GLI3 regulates muscle stem cell entry into GAlert and self-renewal
Source: Nat Commun. 2022 Jul 8;13:3961. doi: 10.1038/s41467-022-31695-5 (PMC9270324; doi:10.1038/s41467-022-31695-5)
Supplement: Supplementary file 3 — Description of Additional Supplementary Files [file 41467_2022_31695_MOESM3_ESM.docx]

**Description of Additional Supplementary Files**

**Supplementary Data 1:** Fold change analysis of transcriptomes from Gli3+/+ ASCs vs Gli3+/+ QSCs.

**Supplementary Data 2:** Fold change analysis of transcriptomes from ASCs Gli3Δ/Δ vs Gli3+/+ QSCs (QSC FC Gli3cKO vs Gli3Ctr) and Gli3Δ/Δ vs Gli3+/+ ASCs (ASC FC Gli3cKO vs Gli3Ctr).
