# Supplementary material for: GLI3 regulates muscle stem cell entry into GAlert and self-renewal
Source: Nat Commun. 2022 Jul 8;13:3961. doi: 10.1038/s41467-022-31695-5 (PMC9270324; doi:10.1038/s41467-022-31695-5)
Supplement: Supplementary file 6 — Reporting Summary [file 41467_2022_31695_MOESM6_ESM.pdf]

nature research

Corresponding author(s): Michael A. Rudnicki

Last updated by author(s): April 8 2022

Reporting Summary

Nature Research wishes to improve the reproducibility of the work that we publish. This form provides structure for consistency and transparency in reporting. For further information on Nature Research policies, see our Editorial Policies and the Editorial Policy Checklist.

Statistics

For all statistical analyses, confirm that the following items are present in the figure legend, table legend, main text, or Methods section.

n/a	Confirmed	
☐	☒	The exact sample size (n) for each experimental group/condition, given as a discrete number and unit of measurement
☐	☒	A statement on whether measurements were taken from distinct samples or whether the same sample was measured repeatedly
☐	☒	The statistical test(s) used AND whether they are one- or two-sided<br>*Only common tests should be described solely by name; describe more complex techniques in the Methods section.*
☒	☐	A description of all covariates tested
☒	☐	A description of any assumptions or corrections, such as tests of normality and adjustment for multiple comparisons
☐	☒	A full description of the statistical parameters including central tendency (e.g. means) or other basic estimates (e.g. regression coefficient) AND variation (e.g. standard deviation) or associated estimates of uncertainty (e.g. confidence intervals)
☐	☒	For null hypothesis testing, the test statistic (e.g. F, t, r) with confidence intervals, effect sizes, degrees of freedom and P value noted<br>*Give P values as exact values whenever suitable.*
☒	☐	For Bayesian analysis, information on the choice of priors and Markov chain Monte Carlo settings
☒	☐	For hierarchical and complex designs, identification of the appropriate level for tests and full reporting of outcomes
☒	☐	Estimates of effect sizes (e.g. Cohen's d, Pearson's r), indicating how they were calculated

Our web collection on statistics for biologists contains articles on many of the points above.

Software and code

Policy information about availability of computer code

Data collection	Bio-Rad CFX96 Maestro instrument (qPCR), Zeiss Axio Observer.D1 (microscopy), EVOS M5000 (microscopy), Zeiss LSM 880 AiryScan (confocal microscopy), Summit (FACS), BD LSRFortessa cell analyzer with BD FACSDiva software v8.0 (flow cytometry), Amnis ImageStream XMk II (imaging flow cytometry), Bio-Rad Image Lab software (Western blot chemiluminescence), Aurora Scientific 300C-LR-FP dual mode muscle lever system (force measurement), Illumina NextSeq 500 system (RNA-sequencing)
Data analysis	R software, GraphPad Prism v7.00, MATLAB-MAthWorks r2015a, MATLAB application SMASH (Semi-Automatic Muscle Analysis using Segmentation of Histology), MuscleJ (ImageJ macro), ImageJ FIJI (imaging), Zeiss ZEN 2 software (microscopy), FlowJo v10 (flow cytometry), IDEAS software (imaging flow cytometry), Bio-Rad Image Lab software (Western blot quantification), Salmon v0.13.1 (RNA-sequencing), DESeq2 v1.28.1 (RNA-sequencing), goseq v1.40.0 (RNA-sequencing), fgsea v1.14.0 (RNA-sequencing)

For manuscripts utilizing custom algorithms or software that are central to the research but not yet described in published literature, software must be made available to editors and reviewers. We strongly encourage code deposition in a community repository (e.g. GitHub). See the Nature Research guidelines for submitting code & software for further information.

Data

Policy information about availability of data

All manuscripts must include a data availability statement. This statement should provide the following information, where applicable:
- Accession codes, unique identifiers, or web links for publicly available datasets
- A list of figures that have associated raw data
- A description of any restrictions on data availability

RNA-sequencing data are available on NCBI GEO accession number [GSE144871] (https://www.ncbi.nlm.nih.gov/geo/). RNA-seq reads were mapped to transcripts from GRCm38_GENCODE.vM19 (https://www.gencodegenes.org/). The raw images for the immunoblots are provided in Supplementary Fig. 9. The source data

underlying Figs. 1a, c, d; 2b, e, f; 3c, e-i, l, m; 4c, d, f, h, j; 5e-h; 6d; 7b-d, f-h and Supplementary Figs. 2d, f, g, i-m; 3b, e, g, i-l, n-q; 4b; 5e, f; 6c; 7a, c-e are provided in the Source Data file. The source data underlying Figs. 5b-d; 6a, b and Supplementary Figs. 1a, b; 5a-c; 6a, b are provided in the Supplementary Data files 1 and 2. All other data supporting the findings of this study are available from the corresponding author on reasonable request.

Field-specific reporting

Please select the one below that is the best fit for your research. If you are not sure, read the appropriate sections before making your selection.

☒ Life sciences ☐ Behavioural & social sciences ☐ Ecological, evolutionary & environmental sciences

For a reference copy of the document with all sections, see nature.com/documents/nr-reporting-summary-flat.pdf

Life sciences study design

All studies must disclose on these points even when the disclosure is negative.

Sample size	Sample size is indicated in the figure legends for each experiment. No sample size calculation was performed. Sample size was determined based on the magnitude and consistency of measurable differences between groups.
Data exclusions	No data were excluded from the analysis.
Replication	Biological and independent replicate experiments were successful. They were replicated independently by at least three of the different co-authors.
Randomization	Gli3-control and Gli3-cKO mice were allocated to muscle regeneration experiments and fiber isolation based on genotype and irrespective of sex. There were no other selection criteria for the allocated animals.<br><br>In vitro cell culture and treatments did not require randomization and were performed using a minimum of 3 biological replicates to avoid any bias.
Blinding	For the injury procedures and in vivo force measurements, researchers were blinded to the genotype. For imaging acquisition, flow cytometry and ex vivo experiments (fiber culture), data analyses were blinded. Researchers performing the imaging acquisition and scoring, the flow cytometry recording and the fiber counting, were unaware of genotype. The genotype was decoded after acquisition and analysis.<br><br>For in vitro cell culture (qPCR, WB) and RNA-sequencing (collecting samples for quiescent and activated satellite cells, myoblasts and myotubes), experiments were not blinded but carried out using standard procedures that should not cause biased results.

Reporting for specific materials, systems and methods

We require information from authors about some types of materials, experimental systems and methods used in many studies. Here, indicate whether each material, system or method listed is relevant to your study. If you are not sure if a list item applies to your research, read the appropriate section before selecting a response.

Materials & experimental systems

n/a	Involved in the study	
☐	☒	Antibodies
☒	☐	Eukaryotic cell lines
☒	☐	Palaeontology and archaeology
☐	☒	Animals and other organisms
☒	☐	Human research participants
☒	☐	Clinical data
☒	☐	Dual use research of concern

Methods

n/a	Involved in the study	
☒	☐	ChIP-seq
☐	☒	Flow cytometry
☒	☐	MRI-based neuroimaging

Antibodies

Antibodies used

Mouse anti-ARL13B [N295B/66] - Abcam ab136648
Mouse anti-α-TUBULIN (acetyl K40) [6-11B-1] - Abcam ab11323
Mouse anti-PAX7 - DSHB PAX7
Mouse anti-GLI3 [6F5] - Gift from Dr. S. Scales (Genentech) N/A
Goat anti-GLI3 - R&D Systems AF3690
Rabbit anti-PKA R2/PKR2 (phospho S99) [E151] - Abcam ab238951
Rabbit anti-IFT88 - Proteintech 13967-1-AP
Mouse anti-TUBULIN (clone DM1A) - Sigma-Aldrich T9026
Mouse anti-GAPDH - UBC AbLab 21-0017
Mouse anti-alpha-ACTIN - Santa Cruz sc-32251

Mouse anti-Myosin Heavy Chain (MyHC) - DSHB MF20
Mouse anti-MYOD1 [5.8A] - Agilent Dako M3512
Mouse anti-MYOGENIN (clone F5D) - Santa Cruz sc-12732
Rabbit anti-DYSTROPHIN - Abcam ab15277
Rat anti-LAMININ [4H8-2] - Sigma-Aldrich L0663
Rabbit anti-LAMININ - Sigma-Aldrich L9393
Chicken anti-SYNDECAN-4 - Gift from Dr. B. Olwin (not commercially available) N/A
Chicken anti-GFP (YFP) - Abcam ab13970
Rabbit anti-GFP (YFP) - ThermoFisher Scientific A-11122
Rabbit anti-phospho-S6 Ribosomal Protein (Ser235/236) [D57.2.2E] - Cell Signaling Technology 4858
Alexa647 mouse anti-Integrin alpha7 (clone R2F2) - UBC AbLab 67-0010-10
Mouse anti-Integrin alpha7-Biotin (clone 3C12) - Miltenyi Biotec 130-102-125
Mouse anti-CD34-Biotin (clone REA383) - Miltenyi Biotec 130-105-830
BV421 mouse anti-SCA1 (clone D7) - BD Biosciences 553108
BV421 mouse anti-CD45 (clone 30-F11) - BD Biosciences 12-0451-83
BV421 mouse anti-CD31 (clone 390) - BD Biosciences 12-0311-81
BV421 mouse anti-CD11b (clone M1/70) - BD Biosciences 12-0112-81
PE-Cy7 mouse anti-CD106 (VCAM1) - BioLegend 105719
PerCP/Cyanine5.5 Streptavidin - BioLegend 405214
Alexa Fluor-conjugated secondary antibodies - ThermoFisher
HRP-conjugated secondary antibodies - Bio-Rad

Validation

All the antibodies were validated in previous published work from the lab and by the manufacturers listed above.
For flow cytometry, the antibody panels used for muscle stem cell sorting (Alexa647 mouse anti-Integrin alpha7 (clone R2F2), anti-Integrin alpha7-Biotin (clone 3C12), Mouse anti-CD34-Biotin (clone REA383), BV421 mouse anti-SCA1 (clone D7), BV421 mouse anti-CD45 (clone 30-F11), BV421 mouse anti-CD31 (clone 390), BV421 mouse anti-CD11b (clone M1/70), PE-Cy7 mouse anti-CD106 (VCAM1), PerCP/Cyanine5.5 Streptavidin) has been validated in the lab previsouly (Addicks et al., NCOMMS, 2019; Sincennes et al., NCOMMS, 2021).
Mouse anti-ARL13B [N295B/66], rabbit anti-IFT88 and mouse anti-α-TUBULIN (acetyl K40) [6-11B-1] have been validated previously to detect the primary cilium in cells as shown in manufacturers' websites.
Mouse anti-PAX7, mouse anti-Myosin Heavy Chain (MyHC), mouse anti-MYOD1 [5.8A], mouse anti-MYOGENIN (clone F5D), rabbit and chicken anti-GFP, mouse anti-GAPDH and mouse anti-TUBULIN (clone DM1A) antibodies have been validated in the lab previously (Addicks et al., NCOMMS, 2019).
Rabbit anti-DYSTROPHIN, rat and rabbit anti-LAMININ [4H8-2] antibodies have been validated in the lab previously (Sincennes et al., NCOMMS, 2021).
Mouse anti-GLI3 [6F5] has been validated by Dr. S. Scales lab (Wen et al., Mol Cell Biol, 2010). Goat anti-GLI3 has been validated for detection of GLI3 as shown in the manufacturer's website.
Rabbit anti-PKA R2/PKR2 (phospho S99) [E151] only detects PKA R2/PKR2 phosphorylated on Serine 99 and has been validated for IF as shown in the manufacturer's website.
Mouse anti-alpha-ACTIN is used as a loading control for western blot and has been validated as shown in the manufacturer's website.
Rabbit anti-phospho-S6 Ribosomal Protein (Ser235/236) [D57.2.2E] detects endogenous levels of ribosomal protein S6 only when phosphorylated at Ser235 and 236 and has been validated as shown in the manufacturer's website.
Chicken anti-SYNDECAN-4 has been validated in the lab (Sincennes et al., NCOMMS, 2021).

Animals and other organisms

Policy information about studies involving animals; ARRIVE guidelines recommended for reporting animal research

Laboratory animals	Mixed genetic background (129SV and C57BL/6) mice were used in the study. All the mice used in this study were males and females, from 2 to 10 month-old. Mice were sex and age-matched in all experiments. The Data source file also provides all this information for the mice used in each experiment.
Wild animals	Study did not involve wild animals.
Field-collected samples	Study did not involve field-collected samples.
Ethics oversight	All experimental protocols for mice used in this study were performed in accordance with the guidelines established by the University of Ottawa Animal Care Committee, which is based on the guidelines of the Canadian Council on Animal Care (CCAC). Protocols were approved by the Animal Research Ethics Board (AREB) at the University of Ottawa.

Note that full information on the approval of the study protocol must also be provided in the manuscript.

Flow Cytometry

Plots

Confirm that:

- [x] The axis labels state the marker and fluorochrome used (e.g. CD4-FITC).
- [x] The axis scales are clearly visible. Include numbers along axes only for bottom left plot of group (a 'group' is an analysis of identical markers).
- [x] All plots are contour plots with outliers or pseudocolor plots.
- [x] A numerical value for number of cells or percentage (with statistics) is provided.

Methodology

Sample preparation	Quiescent satellite cells were obtained from uninjured hindlimb muscles, while activated satellite cells were obtained from CTX-injured tibialis anterior and gastrocnemius muscles 3 days after the induced-injury. Dissected muscles were minced in collagenase/dispase solution followed by dissociation using the gentleMACS Octo Dissociator with Heaters (Miltenyi Biotec to obtain single cell suspensions. Cell doublets were excluded from the analysis (FSC-H vs FSC-A orFSC-W vs FSC-A). Satellite cells (CD31-, CD45-, SCA1-, CD11b-, Itga7+, CD34+/VCAM+) were sorted and used for analysis.
Instrument	Flow cytometry analyses were performed on a BD LSRFortessa cell analyzer and FACS was performed on MoFlo XDP at the Ottawa Hospital Research Institute.
Software	Flow cytometry data acquisition: FACSDiva<br>FACS data acquisition: Summit<br>Data analysis: FlowJo
Cell population abundance	Purity of the post-sort fractions was determined by flow cytometry on the sorted samples. Only samples that were >90% pure were kept for analysis.
Gating strategy	Gating strategy was performed using unstained and single-stained samples. The gating strategy for sorting the satellite cells is provided in a Supplementary Figure 8.

- [x] Tick this box to confirm that a figure exemplifying the gating strategy is provided in the Supplementary Information.
